# Monitoring schistosomiasis risk in East China over space and time using a Bayesian hierarchical modeling approach

**DOI:** 10.1038/srep24173

**Published:** 2016-04-07

**Authors:** Yi Hu, Michael P. Ward, Congcong Xia, Rui Li, Liqian Sun, Henry Lynn, Fenghua Gao, Qizhi Wang, Shiqing Zhang, Chenglong Xiong, Zhijie Zhang, Qingwu Jiang

**Affiliations:** 1 Department of Epidemiology and Biostatistics, School of Public Health, Fudan University, Shanghai 200032, China; 2 Key Laboratory of Public Health Safety, Ministry of Education, Shanghai 200032, China; 3 Laboratory for Spatial Analysis and Modeling, School of Public Health, Fudan University, Shanghai 200032, China; 4 Collaborative Innovation Center of Social Risks Governance in Health,School of Public Health, Fudan University, Shanghai 200032, China; 5 University of Sydney Faculty of Veterinary Science, NSW 2570, Australia; 6 Anhui Institute of Parasitic Diseases, Wuhu, People’s Republic of China 230061, China

## Abstract

Schistosomiasis remains a major public health problem and causes substantial economic impact in east China, particularly along the Yangtze River Basin. Disease forecasting and surveillance can assist in the development and implementation of more effective intervention measures to control disease. In this study, we applied a Bayesian hierarchical spatio-temporal model to describe trends in schistosomiasis risk in Anhui Province, China, using annual parasitological and environmental data for the period 1997–2010. A computationally efficient approach–Integrated Nested Laplace Approximation–was used for model inference. A zero-inflated, negative binomial model best described the spatio-temporal dynamics of schistosomiasis risk. It predicted that the disease risk would generally be low and stable except for some specific, local areas during the period 2011–2014. High-risk counties were identified in the forecasting maps: three in which the risk remained high, and two in which risk would become high. The results indicated that schistosomiasis risk has been reduced to consistently low levels throughout much of this region of China; however, some counties were identified in which progress in schistosomiasis control was less than satisfactory. Whilst maintaining overall control, specific interventions in the future should focus on these refractive counties as part of a strategy to eliminate schistosomiasis from this region.

Schistosomiasis, caused by parasitic blood flukes in the Schistosoma genus^1^, is a zoonotic disease. It remains a serious public health problem worldwide, being endemic mainly in developing countries located in tropical and subtropical regions^2^. According to the World Health Organization, as of 2012 there were at least 249 million people infected and more than 700 million people at risk^3^. The global burden of schistosomiasis has been estimated to be 3.31 (95% confidence interval 1.70–6.26) million disability-adjusted life years (DALYs)^4^. In China, schistosomiasis japonica is responsible for human and animal infections^5^. The disease has a documented history of over 2,100 years along the Yangtze River Basin^6^ and is still regarded as an important parasitic disease. A recent study estimated that there were approximately 185,000 schistosomiasis japonica cases nationwide^7^.

With the implementation of a wide range of schistosomiasis control programs, schistosomiasis prevalence has been greatly reduced^8^ and is currently at a low infection level^9^. However, challenges to control remain and many factors could lead to a resurgence of schistosomiasis, including changes in patient susceptibility to infection and re-infection, effects of global warming, increasing population mobility, and changes in snail habitat and distribution via ecosystem changes as a result of human activities (e.g., the Three Gorges Dams and the South–North Water Conversion Project)^10^,^11^. To eliminate schistosomiasis has proven to be a tough task. National and local surveillance systems have played an important role in schistosomiasis control and elimination^12^,^13^. However, the current surveillance systems predominantly focus on parasitological data collection, such as case reports collected at local anti-schistosomiasis stations (e.g., township) and data aggregated at higher administrative units (e.g., county)^14^,^15^; these data are considered to be inefficient for surveillance. An efficient and timely surveillance system integrating early warning and multi-data streams is needed to improve the implementation and evaluation of targeted control strategies^16^.

Forecasting, as a means of surveillance and early detection, can facilitate the formulation of effective control strategies for schistosomiasis. Time series analysis^17^,^18^,^19^ and its derivatives^20^ are currently the predominant approaches. Although these approaches consider the temporal dimension they neglect the geographical clustering characteristic of schistosomiasis. Some researchers^21^ in China have forecasted the risk of S. japonicum infection by modeling the temporal and spatial dimensions separately, ignoring the spatio-temporal interaction dimension. In contrast, in the current study we built a hierarchical Bayesian (HB) spatio-temporal model to investigate the dynamic pattern of human schistosomiasis risk in Anhui Province, East China, a typical schistosomiasis-endemic area along the Yangtze River Basin. First, we described schistosomiasis risk in Anhui using annual county-level parasitological data for the period 1997–2010, and briefly discuss the HB spatio-temporal model and modeling approaches. We then used the best predictive model to forecast schistosomiasis risk for the period 2011–2014. In closing, we discuss the potential implications of our findings for schistosomiasis control.

## Results

**Figure 1:**
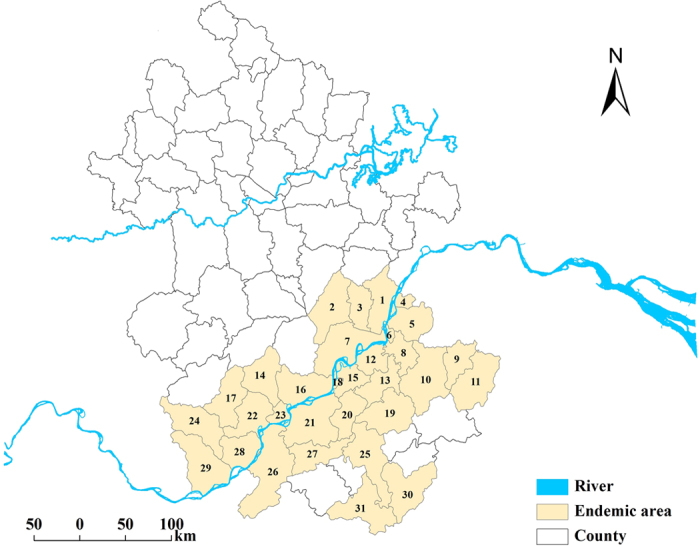
Endemic area of schistosomiasis japonica in Anhui Province, People’s Republic of China. The river in the north is the Huaihe River and the one in the south is the Yangtze River. The number appearing in the map is the county ID. The map was created using ArcGIS software (version 10.0, ESRI Inc. Redlands, CA).

**Figure 2:**
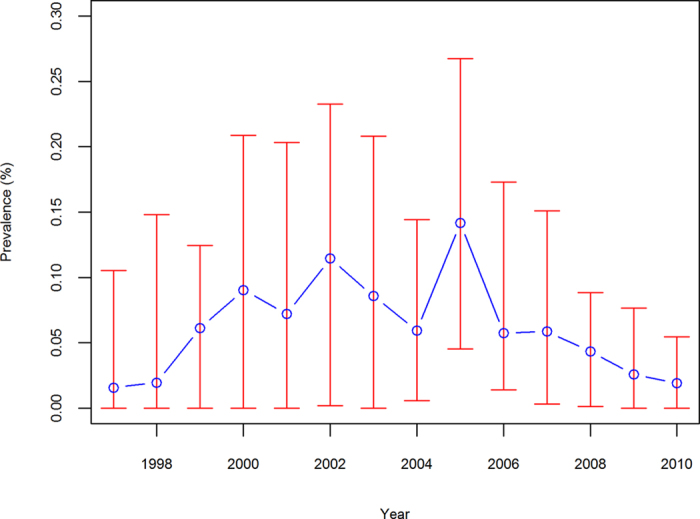
Prevalence of *S. japonicum* infection for endemic counties in Anhui Province, China, from 1997 to 2010. The red vertical lines denote interquartile range and the blue circles denote the median.

Figure 1 shows the endemic area of schistosomiasis japonica within our study region. Figure 2 depicts the change in annual prevalence of schistosomiasis risk during the study period. The median prevalence showed an increasing trend from 0.016% in 1997 to 0.115% in 2002; it then decreased gradually to 0.019% in 2010. There was a resurgence in prevalence (0.142%) in 2005. A higher prevalence was accompanied by a wider interquartile range (IQR). Overall, the schistosomiasis risk fluctuated through the study period.

**Figure 3:**
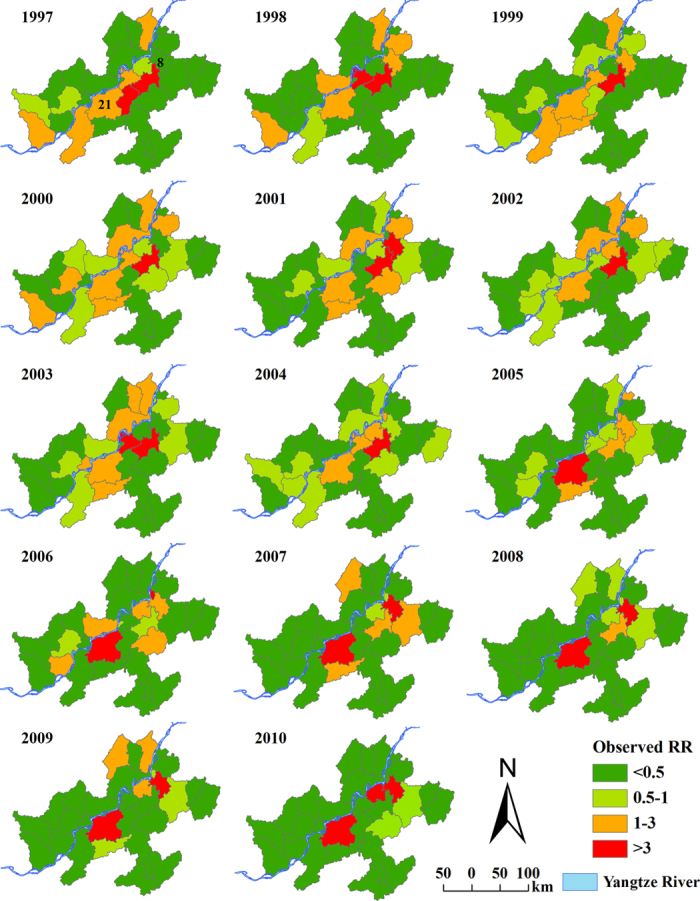
Annual observed relative risk (RR) of schistosomiasis in Anhui Province, China, from 1997 to 2010. The maps were created using ArcGIS software (version 10.0, ESRI Inc. Redlands, CA).

Figure 3 is a map of observed relative risk (RR) of schistosomiasis during the period 1997–2010. The spatial pattern of the disease risk was different across the study years. High levels of schistosomiasis risk (i.e., RR >1) were observed mainly in areas along the Yangtze River; areas of low levels of disease risk were located away from the river, mainly in the south and east parts of the study region. Some counties presented high and increasing risks during the study period, such as county 21 (county IDs are shown in Fig. 1) with RRs between 1 and 3 during 1997–2004 and rising to >3 there afterwards. Other counties–for example county 8–showed a fluctuating risk during 1997–2006 and then maintained a high relative risk during the remaining years. Geographically, the spatial extent of high risk decreased and became focal during the study period.

We used a dynamical spatio-temporal model (denoted as *m*_1_) to study the trends in schistosomiasis risk in the study region. For a comparison, we also specified a non-dynamical spatio-temporal model (denoted as *m*_2_), a spatial model (denoted as *m*_3_), and a temporal model (denoted as *m*_4_). In addition, we evaluated these models’ zero-inflated version. For model selection, all the models were fit to RRs of schistosomiasis for the period 1997–2008 (*T* = 12). A comparison was made between observed RR values of the last two years (2009 and 2010) and RRs predicted by the models at *T* + 1 and *T* + 2. For each of these evaluations, we calculated the mean squared predictive errors (MSPEs), which measure model accuracy. In addition, the deviance information criterion (DIC)^22^ was considered.

The MSPE and DIC results of all models are presented in Table 1. As indicated by MSPEs, the zero-inflated models performed better than their ordinary model counterparts. With the zero-inflated negative binomial (NB) likelihood, the dynamical spatio-temporal model (*m*_1_) outperformed other models, with the smallest MSPEs of 3.96 in 2009 and 0.85 in 2010. Hence, model *m*_1_ with a zero-inflated NB likelihood was selected as the best predictive model (BPM) for forecasting. Interestingly, this model also fitted the data best as indicated by the smallest DIC.

Table 2 shows the results of the BPM using data for the period 1997–2010. Temperature and rainfall were not significantly associated with risk. Distance to the Yangtze River was negatively correlated with schistosomiasis risk. The schistosomiasis risk decreased over time but this change was not statistically significant. The posterior mean of the overdispersion parameter (*r*) was 2.56 and the 95% credible interval (CI) was 1.86 to 3.43, namely the overdispersion parameter was significantly greater than zero. The posterior mean of the zero-inflated parameter *α* was also significantly different from zero (posterior mean 2.36, 95% CI 1.59 to 3.35). The posterior mean of the transition parameter *ρ* was 0.91 (95% CI 0.86 to 0.95), indicating a stationary temporal process. The posterior mean of the variance parameter *κ*^2^ was 0.24 (95% CI, 0.15, 0.36).

**Figure 4:**
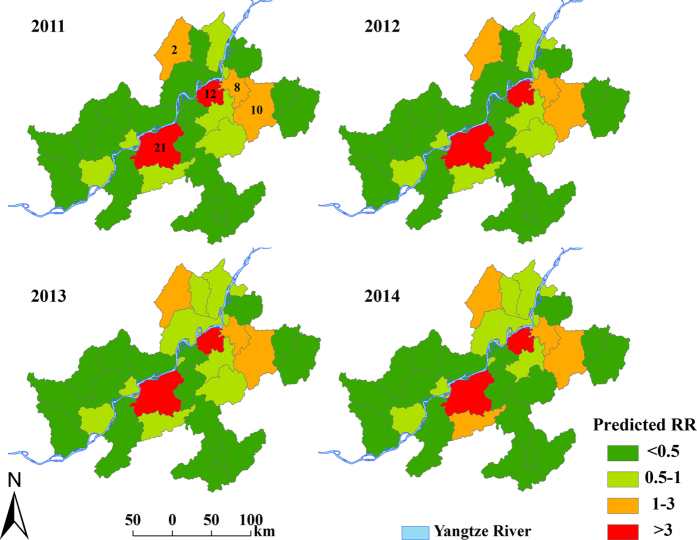
Annual predicted relative risk (RR) of schistosomiasis in Anhui Province, China, from 2011 to 2014. The maps were created using ArcGIS software (version 10.0, ESRI Inc. Redlands, CA).

**Figure 5:**
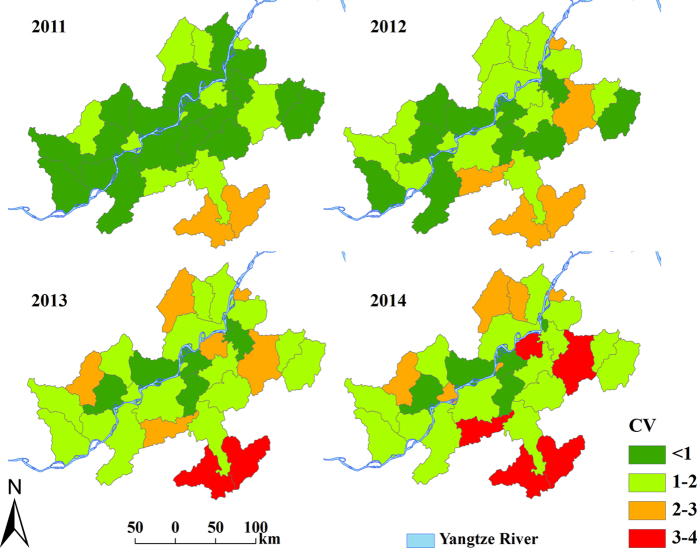
Annual coefficient of variation of predicted relative risk (RR) for schistosomiasis in Anhui Province, China, from 2011 to 2014. The maps were created using ArcGIS software (version 10.0, ESRI Inc. Redlands, CA).

Figure 4 displays the annual map of predicted RRs of schistosomiasis for the period 2011–2014. Most of the endemic counties had RRs <1, indicating relatively low schistosomiasis risk. Counties 21 and 12 had the highest risk (RR >3) and counties 8, 10, and 2 were also high-risk areas (RR ranging from 1 and 3). Figure 5 presents corresponding coefficients of variation (CV) of the forecasting. As expected, the maps present a rising trend over time: a higher level of uncertainty in forecasting occurs over time.

## Discussion

The purpose of this study was to explore the spatio-temporal dynamics of schistosomiasis risk in Anhui Province, China, and then to forecast risk using a Bayesian spatio-temporal model with a computationally efficient and statistically powerful inference approach. In this approach, it is assumed that observations are determined by a hidden latent process, which is important for understanding the etiology of a phenomenon^23^. Our results identified the spatio-temporal pattern of schistosomiasis risk after adjusting for some important environmental factors and the forecasting risk maps provided useful information for future schistosomiasis monitoring and control.

The spatial variation of schistosomiasis risk and the association between environmental factors and the disease can be explained by the known biology of schistosoma and freshwater snails (i.e., *O.hupensis*). Higher temperature and more rainfall are conducive to the parasite’s life cycle and formation of snail habitat. However, abnormal weather conditions can have negative effects on both the parasite and the snail^24^,^25^. During 1997–2010, temperature in the region was very low in 2005 and rainfall was extremely heavy in 2009. This might explain why temperature and rainfall were not significantly positively associated with schistosomiasis risk in our analysis. We found that distance to the Yangtze River was significantly negatively correlated with disease risk, likely because of an increased risk of contacting infected water via swimming, fishing and agricultural activities. Of note, there was some spatio-temporal random effect (*η*_*it*_ in formula [3]) that could not be explained by the fixed variables. The estimate of transition parameter *ρ* shows that formula 3 is stationary, indicating that the small-scale spatio-temporal variation of schistosomiasis risk was stable over time, i.e. it was not explosive or decaying.

Schistosomiasis disease risk tended to decrease over time but the change was not statistically significant. The study area was subject to two important, national schistosomiasis control programs during the period 1997–2010. The first one was a 10-year World Bank Loan Project (WBLP) launched in 1992, largely based on large-scale chemotherapy. The second was a revised control strategy which was initiated in 2005, using integrated measures and an emphasis on controlling the source of infection. In addition, each county used available funds to undertake individual control strategies^14^. As the drug-based strategy is a host-targeted treatment, it can only control the disease to a certain extent. Studies^26^,^27^ have shown that schistosomiasis risk rebounded shortly after the WBLP was completed in 2001. Our study shows that the median prevalence reached a peak in 2002 (Fig. 2). The revised strategy focused largely on interrupting the life cycle of the parasite^28^,^29^. An important measure was to replace water buffalo (a prevalent infection source in the lake and marshland area) with agricultural machinery. A reduced median prevalence with narrowed IQR after 2005–demonstrated in our results (Fig. 2)–suggests that the latter program was likely more effective. A limitation of the revised control strategy is that the intervention measures cannot completely block the life cycle of the parasite since more than 40 species of mammals^30^ can serve as potential zoonotic reservoirs, in addition to water buffalo and humans. Infected *O. hupensis* snails were still found in certain locations along the Yangtze River^31^. These two features might prevent further reduction of schistosomiasis risk.

The focus of our study was to forecast schistosomiasis risk. As shown in Fig. 4, the risk for some counties remained elevated (i.e., RR >1; for example, counties 21, 12 and 8); these should be priority areas for the implementation of control measure. Unexpectedly, historically low-risk counties (counties 10 and 2 in this study) became high risk counties; these require close monitoring in a disease control program. The rising uncertainties in the forecasting of disease risk over time (Fig. 5) indicates that the accuracy of risk prediction may be reliable for a short-term forecast but not for a long-term forecast, which may limit its utility for decision-making in some circumstances. Such uncertainties need to be carefully assessed when interpreting maps for disease control^32^.

We included the spatio-temporal dependence in formula 3 that shows how the current value *η*_*it*_ behaves given the behavior of “nearby” current *η*_*jt*_ (*j* are spatial neighborhoods of *i*) and the most recent past values *η*_*i*,*t* − 1_. This model characterizes the evolution of schistosomiasis risk over space and time. Another way to model the spatio-temporal dependence is to build a moment-based model (e.g., Kriging approach) and it has been used in some previous schistosomiasis risk studies^33^,^34^. The latter approach ignores the directionality of time and specifies only the first and second moments of *η*_*it*_ in the temporal domain of interest. It can ignore the genesis of the temporal dependence and generally performs much worse in forecasting^35^. We also made a comparison with a non-dynamical spatio-temporal model, a purely spatial model, and purely temporal model. MSPE results justified our adoption of the dynamical spatio-temporal model. DIC results also indicated that the dynamical spatio-temporal model is the best fitting model.

The spatio-temporal model accounted for overdispersion to a large extent, which has seldom been considered in previous studies of schistosomiasis risk forecasting. Instead of the standard Poisson distribution to model the potential extra-Poisson variation we assumed the number of infected individuals in each county follows a NB distribution. Additionally, a large proportion of individuals were considered to be non-infected due to heterogeneities in environmental exposure^36^ and imperfect diagnostic approaches for schistosomiasis^37^ (see Fig. 2). The zero-inflated parameter of the zero-inflated NB model (*α* = 2.36, 95% CI 1.59 to 3.35) was significantly different from zero suggesting that there were a significant number of zeros in the observed data that could not be included in the NB distribution. Besides the statistical consideration, the zero-inflation model is epidemiologically used for better understanding the presence and absence of infection in the study area and the count process accounts for the level (or strength) of the risk of infection. The MSPE and DIC (Table 1) results indicated that the zero-inflated NB model improved the performance for both data fitting and risk forecasting.

Some limitations of this study deserve discussion. First, only a few environmental factors (i.e., temperature, rainfall, and the Yangtze River) were included in our study, whilst socio-economic and socio-behavioral indicators–which might have an important impact on schistosomiasis risk as well as the effectiveness of targeted control measures–were absent. Second, the specificity of serological assays and the sensitivity of stool examination tests are not perfect^38^ and this uncertainty was not considered in our modeling. Modeling with diagnostic errors should be considered in future studies. Finally, we were not able to directly attribute the spatio-temporal changes in schistosomiasis risk to the two national control strategies and the environmental factors in the current analysis. Further studies are needed to understand the observed trend in disease risk.

In summary, we proposed a dynamical spatio-temporal model based on a hierarchy approach to forecast schistosomiasis risk in this study. Our model disclosed the spatio-temporal dynamics of schistosomiasis risk and the forecasting results identified three stable high-risk counties and two potential high-risk counties, which should be the priority areas for targeted interventions. The forecasting maps provide an empirical basis for monitoring schistosomiasis risk in the study area.

## Methods

### Study area

Anhui Province, located across the basins of the Huaihe River (in the North) and the Yangtze River (in the South), spans approximately 139,600 km^2^ in east China and has a population of 60.83 million (2014). The province is characterized by a range of landscapes: plains in the northern and north-central parts and mountains and a series of hills in the south-western and south-eastern parts. The province has a subtropical humid monsoon climate and “plum” rains occur in June and July and may cause flooding, which is conducive to growth of *Oncomelania hupensis*, the intermediate host of schistosomiasis japonica.

### Parasitological data

The county-level schistosomiasis data were obtained from cross-sectional surveys conducted by the health professionals of the Anhui Institute of Parasitic Diseases repeated annually between 1997 and 2010. For each year, parasitological data were originally collected through village-based field surveys of randomly selected villages, using a two-pronged diagnostic approach (all residents aged 5 to 65 years were screened by a serological test and then confirmed by a fecal parasitological test [Kato-Katz technique]^39^, then were reported to township and finally to county. During our study period, the yearly numbers of sample villages and individual participants ranged between 1,610 and 2,000, and 1,045,708 and 1,575,012, respectively. For our analysis, we removed counties that had no infected individuals during the study period. Thirty-one counties were included in this study (Fig. 1).

### Environmental data

Schistosomiasis is a well-known environment-related disease, the transmission of which is strongly associated with temperature, rainfall and access to water^40^,^41^,^42^. In our study, environmental data included rainfall, temperature and distance to the Yangtze River. Monthly rainfall and temperature data during the period 1997–2010 were obtained from the China Meteorological Data Sharing Service System (http://cdc.cma.gov.cn/home.do). With available data at 756 meteorological stations in China, inverse distance weighting (IDW) interpolation was used to derive estimates within the study area. ArcGIS software (version 10.0, ESRI Inc.; Redlands, CA, USA) was used to extract monthly-average rainfall and temperature for each county. Data on the Yangtze River were downloaded from Conservation Science Data Sets of World Wildlife Foundation at http://worldwildlife.org. For each county, the Euclidian distance from the centroid of the county to the Yangtze River was calculated using ArcGIS software.

### Ethical statement

Approval for oral consent and other aspects of the surveys were granted by the Ethics Committee of Fudan University (ID: IRB#2011-03-0295). Written informed consent was also obtained from all participants.

### Statistical analysis

#### Hierarchical spatio-temporal model structure

Assume that there is a true unobserved spatio-temporal process hidden behind the yearly counts of schistosomiasis cases, which is incorporated into the framework of an HB statistical model. The spatial domain is discrete and consists of the 31 counties in the study area. Consequently, the size of count data we model is fixed at 31. The basic representation of the HB model is typically composed of three levels^23^, namely the data level (whose conditional probability distribution given processes and parameters is independent), the process level (which determines change of data level given parameters), and the parameter level (which exists in the previous levels).

### Data level

Let *Z*_*it*_ denotes the number of infected individuals at time *t*, in county *i*, where *i* = 31 and *t* = 14. Specifically, the data level is assumed to be a product of independent negative binomial (NB) distributions considering the possible overdispersion in the observed counts. That is,


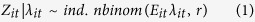


where *Z*_*it*_ and *E*_*it*_ are the observed number of schistosomiasis cases and the expected number of schistosomiasis cases in the county *i* at time *t*, respectively, and *r* is the overdispersion parameter. *Z*_*it*_ approaches a Poisson distribution as *r* approaches zero. Although NB models may be ideal for over-dispersion, they may not be suitable when the data include too many zero values^43^. Since the schistosomiasis data in our analysis are counts characterized by many zeros, we evaluated the NB distribution as well as its zero-inflated version. *λ*_*it*_ is the underlying RR at county *i* and time *t*, and it is the hidden process of interest. The conditional distribution of *λ*_*it*_ given parameters, i.e. the process level, is where the spatio-temporal dependence can be modeled.

### Process model

The process model is:


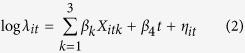


where *X*_*itk*_ are fixed covariates that are specified as the environmental factors and *β*_*k*_ are corresponding coefficients; time *t* (and its corresponding coefficient *β*_4_) is used as an ordinal variable. *X*_*itk*_ and *t* capture the large-scale spatio-temporal variation of schistosomiasis risk while *η*_*it*_ captures the small-scale variation which explains any spatio-temporal statistical dependence. The marginal spatial dependence is modeled according to an intrinsic conditional autoregressive (CAR) model^44^ and the temporal dependence is modeled as a Gaussian first-order autoregressive (i.e., AR(1)) process^45^:


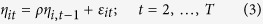


where *ρ* is the transition parameter with |*ρ*| < 1 in case of stationarity and the instantaneous spatial correlation, *ε*_*it*_, is independent from *η*_*i*,*t* − 1_ and is distributed according to the Gaussian Markov random field,


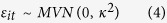



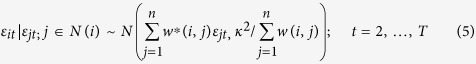


where *κ*^2^ is the variance parameter at time *t, N*(*i*) denotes spatial neighborhoods of county *i* (here we assume that the neighborhood structure does not change over time), and *w*(*i, j*) is a weights matrix element and w^*^(*i, j*) is a standardized form of a weights matrix, defining the relationship between county *i* and its neighbor county *j*. The weight is defined simply as *w*(*i, j*) = 1 if the two counties are adjacent (i.e., share a common border) and *w*(*i, j*) = 0 otherwise. As formula 3 shows how the current value *η*_*it*_ is related mechanistically to its past value and its current spatial neighbors, we call it a dynamical spatio-temporal model.

### Parameter level

At this level, we specify the joint prior distribution for the parameters in data level and process level to complete the HB model. The parameters include the overdispersion parameter {*r*}, the fixed-effect coefficients *β* = (*β*_1_, *β*_2_, *β*_3_, *β*_4_), the transition parameter {*ρ*}, and the variance parameter {*κ*^2^}. Recall that we also consider a zero-inflated version of NB likelihood at the data level, hence a parameter (denoted as {*α*}) that deals with extra zeros that are not generated by the NB distribution is also included (details about *α* can be found in Appendix A). Assuming independence and using [*Y*] as generic notation for the density of *Y*, we have


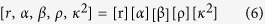


where the prior distributions of individual parameters are specified as follows:


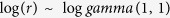



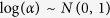



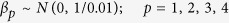



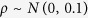



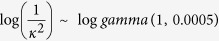


Note that *α* is excluded from (6) for a NB likelihood.

### Model inference

Instead of using Markov Chain Monte Carlo (MCMC) methods, which are traditionally relied on in Bayesian computation, we employ the integrated nested laplace approximation (INLA) approach as the model inference method. Proposed by Rue and Martino^46^, INLA is an approach to perform fast and efficient Bayesian inference through the accurate approximation to the marginal posterior densities of (hyper) parameters and latent variables in latent Gaussian models. The advantage of INLA over MCMC is that INLA uses an approximation for inference and hence avoids the intense computational demands, convergence, and mixing problems sometimes encountered by MCMC algorithms^46^. More details about this approach and its applications can be found elsewhere^46^,^47^,^48^. All model fitting was performed using R software (R Development Core Team 2013), specifically the R-INLA package.

The methods were carried out in accordance with the approved guidelines in this journal.

## Additional Information

**How to cite this article**: Hu, Y. *et al*. Monitoring schistosomiasis risk in East China over space and time using a Bayesian hierarchical modeling approach. *Sci. Rep.* **6**, 24173; doi: 10.1038/srep24173 (2016).

## Supplementary Material

Supplementary Information

## Figures and Tables

**Table 1:** Mean squared predictive error (MSPE) and deviance information criterion (DIC) values for all models of schistosomiasis in Anhui Province, China, 1997 to 2010, tested.

Data level	Process level	MSPE_1_	MSPE_2_	DIC
NB	*m*_1_	7.104	1.654	2827.525
	*m*_2_	172.616	2.228	3126.857
	*m*_3_	130.846	1.655	2984.216
	*m*_4_	173.343	2.228	3126.893
Zero-inflated NB	*m*_1_	3.964	0.853	2812.538
	*m*_2_	68.279	2.178	3006.359
	*m*_3_	24.484	1.644	2906.787
	*m*4	122.009	2.212	3055.228

NB: negative binomial distribution; MSPE_1_: mean squared predictive error at 2009; MSPE_2_: mean squared predictive error at 2010.

**Table 2:** Posterior estimates (mean, 95% credible interval, and median) of model parameters with zero-inflated negative binomial distribution of a model of schistosomiasis risk in Anhui Province, China, 1997 to 2010.

Parameters	Mean	*Q*_0.025_	*Q*_0.975_	Median
Temperature	0.4e-03	−3.5e-03	4.3e-03	0.4e-03
Rainfall	0.1e-03	−0.1e-03	0.2e-03	0.1e-03
The Yangtze River	−0.023	−0.037	−0.010	−0.023
Time	−0.027	−0.055	0.001	−0.026
*r*	2.562	1.857	3.432	2.533
*α*	2.355	1.587	3.348	2.316
*ρ*	0.913	0.863	0.950	0.915
*κ*^2^	0.237	0.148	0.357	0.232

*r*: overdispersion parameter in the negative binomial distribution; *α*: the zero-inflated parameter; *ρ*: the transition parameter; *κ*^2^: the variance parameter in the instantaneous spatial effect.
