# Supplementary material for: Monitoring schistosomiasis risk in East China over space and time using a Bayesian hierarchical modeling approach
Source: Sci Rep. 2016 Apr 7;6:24173. doi: 10.1038/srep24173 (PMC4823756; doi:10.1038/srep24173)
Supplement: Supplementary Information [file srep24173-s1.pdf]

Monitoring schistosomiasis risk in East China over space and time using a Bayesian hierarchical modeling approach

Yi Hu[1,2,3], Michael P. Ward[4], Congcong Xia[1,2,3], Rui Li[1,2,3], Liqian Sun[1,2,3], Henry Lynn[1,2,3], Fenghua Gao[5], Qizhi Wang[5], Shiqing Zhang[5], Chenglong Xiong[1,2], Zhijie Zhang[1,2,3,*], Qingwu Jiang[1,2,3]

[1] Department of Epidemiology and Biostatistics, School of Public Health, Fudan University, Shanghai 200032, China
[2] Key Laboratory of Public Health Safety, Ministry of Education, Shanghai 200032, China
[3] Laboratory for Spatial Analysis and Modeling, School of Public Health, Fudan University, Shanghai 200032, China
[4] University of Sydney Faculty of Veterinary Science, NSW 2570, Australia
[5] Anhui Institute of Parasitic Diseases, Wuhu, People's Republic of China 230061, China

* Corresponding author: Zhijie Zhang (epistat@gmail.com)

Supplementary Information

In a zero-inflated negative binomial distribution, we allow for additional probability of Y(s, t) = 0. For example, under the Type 2 formulation in INLA, we have

$$P[Y(i,t) = k] = p(i,t)I_{k=0} + [1 - p(i,t)] * NB[k, r, \theta(i,t)]$$

where $p(i,t)$ is the extra probability added to the negative binomialfor k = 0, I is an indicator function (i.e., I equals 1 if k=0 and zero otherwise), $r$is the overdispersion parameter, and $\theta(i,t)$is the probability that a randomly selected individual in county *i* and time *t* is infected. The extra probability ofzero, $p(i,t)$, depends on the linear predictor η_{it} (i.e., the extraprobability depends on the covariates and the spatio-temporal random effect). That is

$$p(i,t) = 1 - (\frac{\exp[\eta_{it}]}{1 + \exp[\eta_{it}]})^{\alpha}$$

This means that the probability of zero infected individuals at county *i* at time *t* is inversely proportional to the linear predictor η_{it}. We also have $\pi(i,t) = \exp[\eta_{it}] / \{1 + \exp[\eta_{it}]\}$ proportionaltoη_{it}, and writing this in one equation, we arrive at

Type 2: $P[Y(i,t) = k] = [1 - \pi(i,t)^{\alpha}]I_{k=0} + [\pi(i,t)^{\alpha}] * NB[k, r, \theta(i,t)]$

The zero-inflated parameter α can be thought of as a parameter that controlsboth the impact of the linear predictor on the extra probabilityat zero as well as that for the positive counts. When $\alpha = 0$ we have$\pi(i,t)^{\alpha} = 1$ and simply $Y(i,t)$ follows a negative binomial distribution, namely, no extraprobability at zero. As $\pi(i,t)^{\alpha} \in (0,1)$ and $\alpha > 0$, $\pi(i,t)$ increases andthe extra probability of zero decreasesif αincreases.

There are three types of zero-inflated negative binomial distribution functions in INLA, namely, Type 0, Type 1, and Type 2 formulation. All these formulations are fitted, but MSPE results showed that Type 2 outperformed Type 0 and Type 1, which are as follows:

Type 0: $P[Y(i,t) = k] = p(i,t)I_{k=0} + [1 - p(i,t)] * NB[k, r, \theta(i,t)]$

Type 1: $P[Y(i,t) = k] = p(i,t)I_{k=0} + [1 - p(i,t)] * NB[k, r, \theta(i,t)]$
